# Supplementary material for: EC-Bench: a benchmark for enzyme commission number prediction
Source: Bioinform Adv. 2026 Jan 8;6(1):vbag004. doi: 10.1093/bioadv/vbag004 (PMC12889163; doi:10.1093/bioadv/vbag004)
Supplement: vbag004_Supplementary_Data [file vbag004_supplementary_data.zip › sunburst_plots/f1_score_comparison_deepectransformer-regular_vs_enzbert-regular.html]

F1 Score & Frequency Comparison

## Comparison: deepectransformer-regular vs enzbert-regular

### Threshold: 100

### Threshold: 30

#### Frequency Distribution
