# Supplementary material for: EC-Bench: a benchmark for enzyme commission number prediction
Source: Bioinform Adv. 2026 Jan 8;6(1):vbag004. doi: 10.1093/bioadv/vbag004 (PMC12889163; doi:10.1093/bioadv/vbag004)
Supplement: vbag004_Supplementary_Data [file vbag004_supplementary_data.zip › sup-material-final.pdf]

Supplementary Materials

EC-Bench: A Benchmark for Enzyme Commission Number Prediction

Saeedeh Davoudi, Christopher S. Henry, Christopher S. Miller, and Farnoush Banaei-Kashani

Supplementary Tables

Table S1. Parameters of the MLP classifier used for stacking training.

Parameter	Value	Description
Hidden layer size	100	One hidden layer containing 100 neurons.
epochs	200	Number of iterations (epochs) for training the model.
alpha	0.001	Regularization parameter (L2 penalty) to prevent overfitting.
Learning rate	Adaptive, initial value: 0.001	Adjusts the learning rate dynamically, reducing it when loss plateaus.

Table S2. EnzBert finetuning parameters.

Parameter	Value / Description
Base model	ProtBert-BFD pretrained on protein sequences from BFD
Dropout on (CLS)	0.2
Batch size	2
Accumulation step	16
Learning rate	1.0×10^{-5}
Optimizer	Adam ($\beta_1 = 0.9$, $\beta_2 = 0.999$)
Lr scheduler	Lr (epoch) = 0.8 × Lr (epoch − 1)
Number epochs	15

Table S3. ProteinBERT training parameters.

Parameter	Value / Description
Pretraining dataset	~106 million protein sequences from UniRef-90
Model variants	Supports sequence lengths of 128, 512, and 1024 tokens
Architecture style	Inspired by BERT, with 6 transformer blocks, combining local (convolutional/FC) and global (attention) pathways
Global attention	Uses linear-complexity global-attention layers enabling support for very long sequences
Parameter count	~16 million parameters, significantly smaller than similar models
Pretraining tasks	Masked language modeling + Gene Ontology (GO) annotation prediction
Activation function	GELU
Optimizer	Adam
Number of epochs	~6.4 passes over the dataset (28 days of training)
Hardware/training speed	~280 protein records per second on an Nvidia Quadro RTX 5000 GPU
Fine-tuning strategy	Freeze all layers + train classification head (40 epochs), then unfreeze full model (40 additional epochs), finishing with 1 epoch at longer sequence length
Fine-tuning duration per task	~14 minutes on a single GPU, across 9 benchmarks

Supplementary Figures

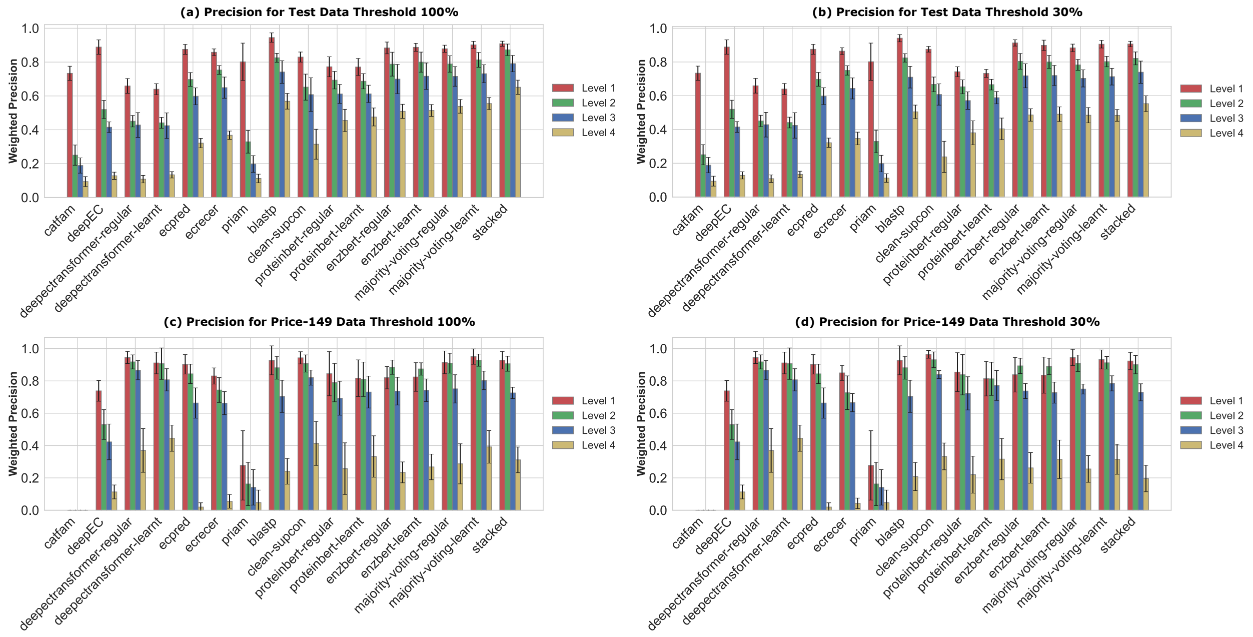

Figure S1. Weighted precision for exact EC number prediction across all models and test sets.

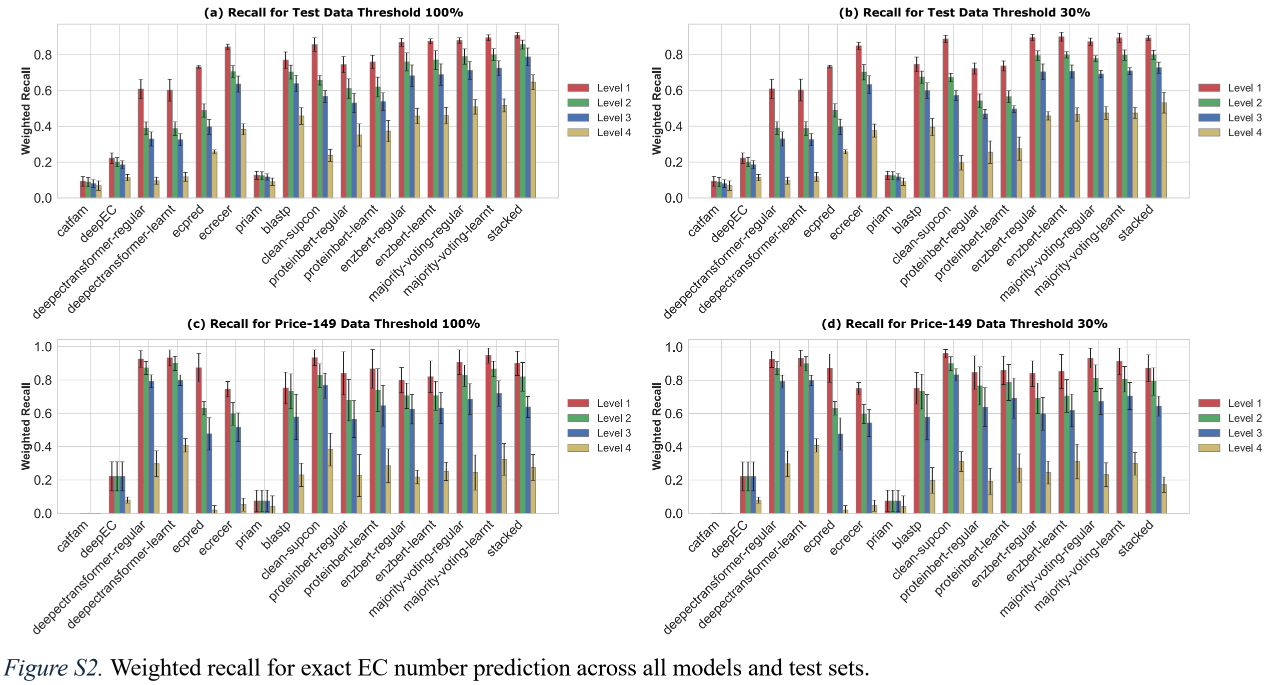

Figure S2. Weighted recall for exact EC number prediction across all models and test sets.

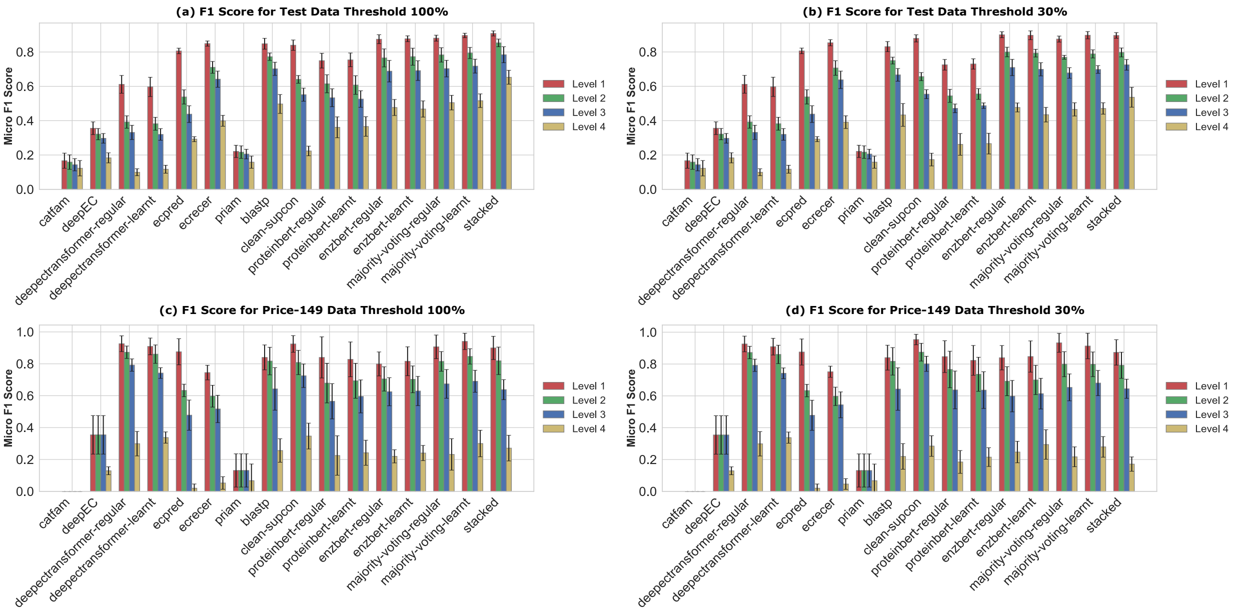

Figure S3. Micro F1 scores for exact EC number prediction across all models and test sets.

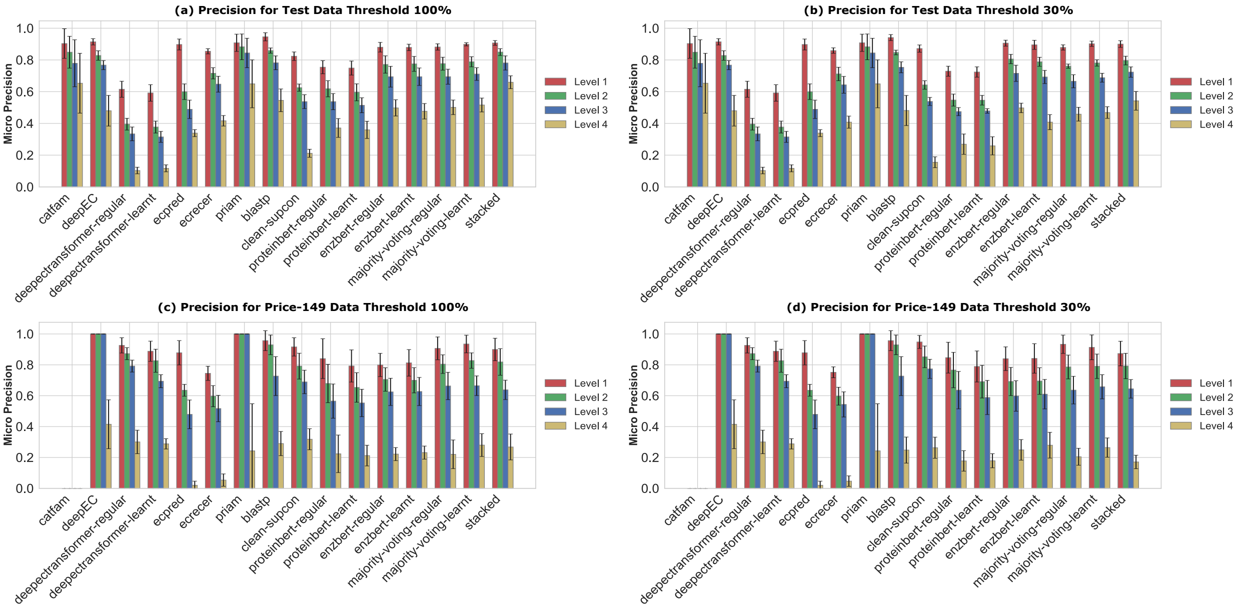

Figure S4. Micro precision for exact EC number prediction across all models and test sets.

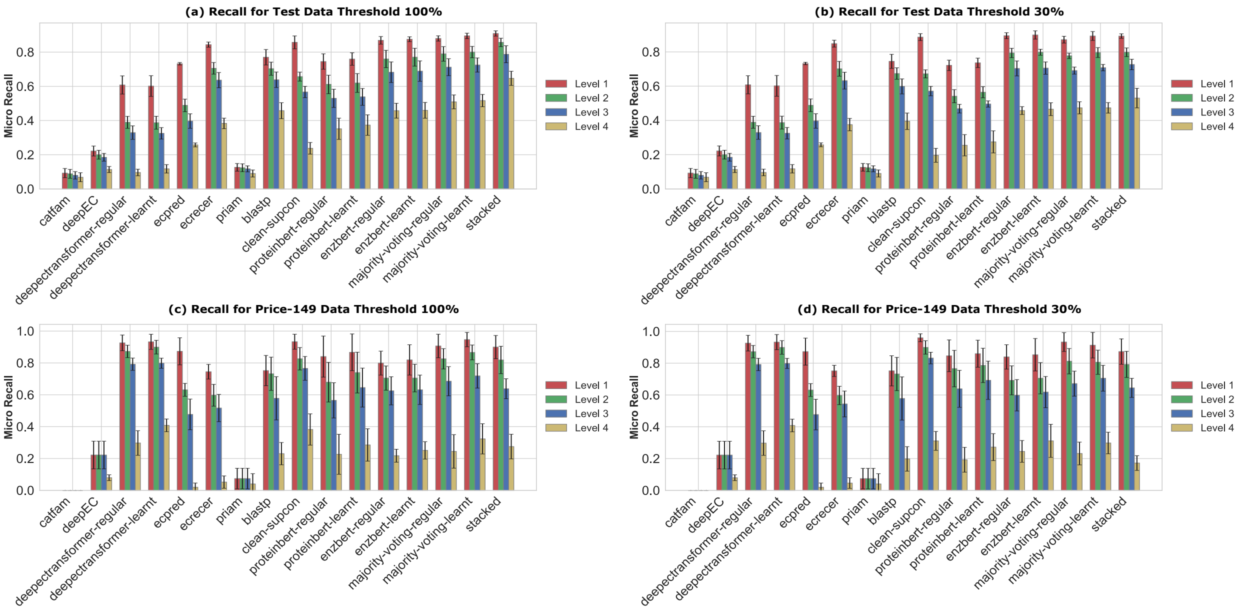

Figure S5. Micro recall for exact EC number prediction across all models and test sets.

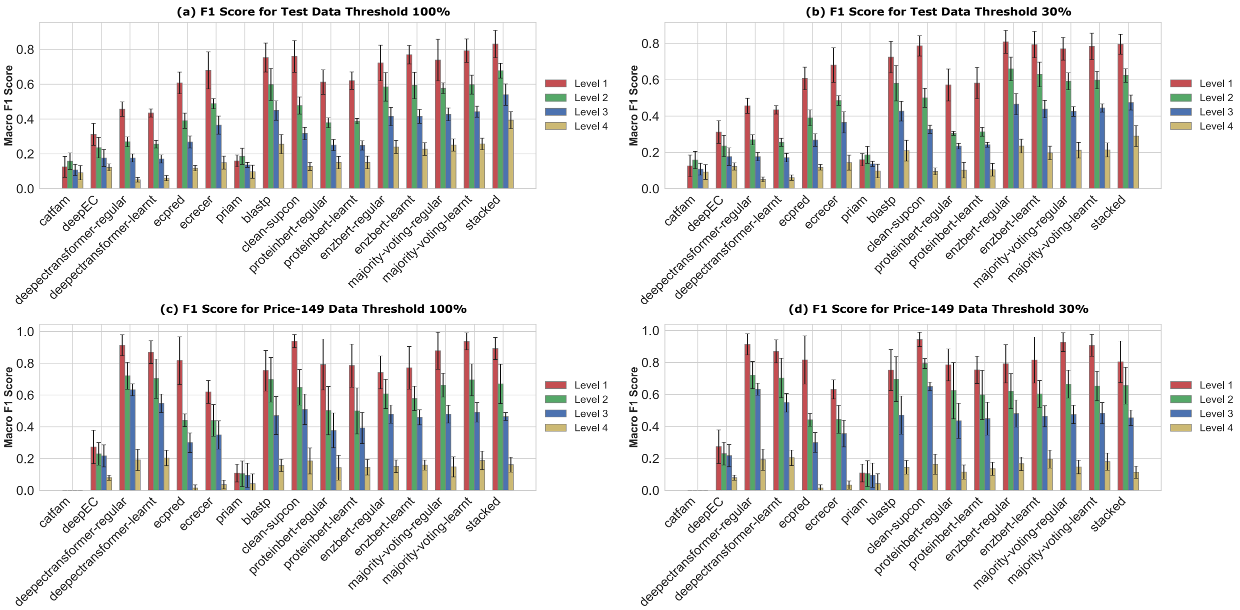

Figure S6. Macro F1 scores for exact EC number prediction across all models and test sets.

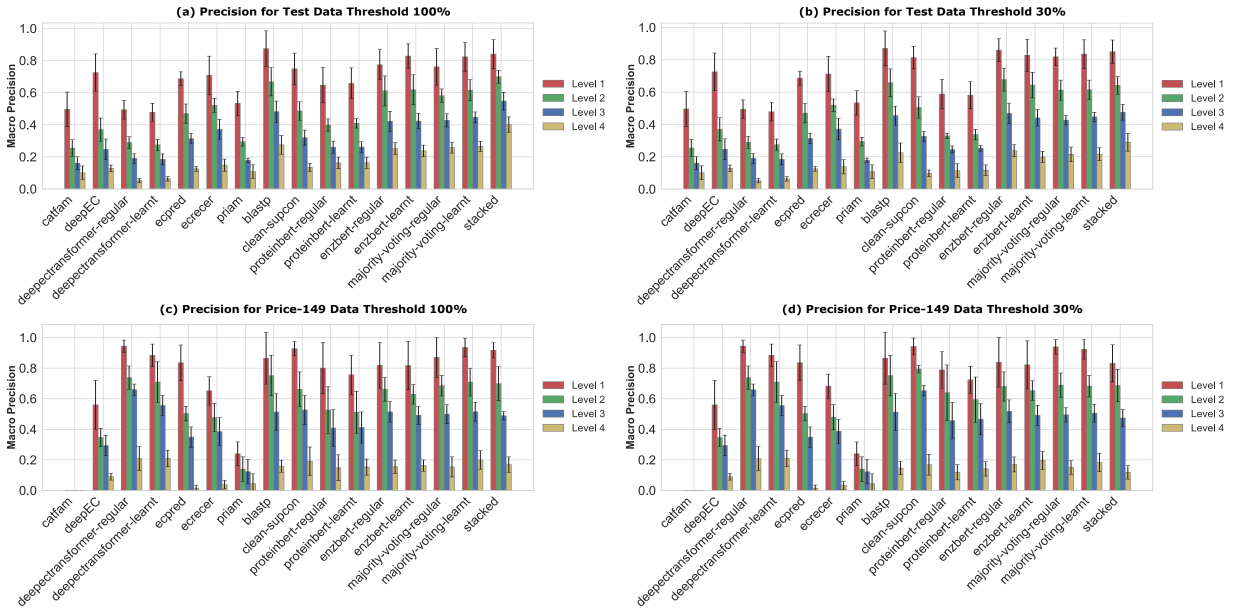

Figure S7. Macro precision for exact EC number prediction across all models and test sets.

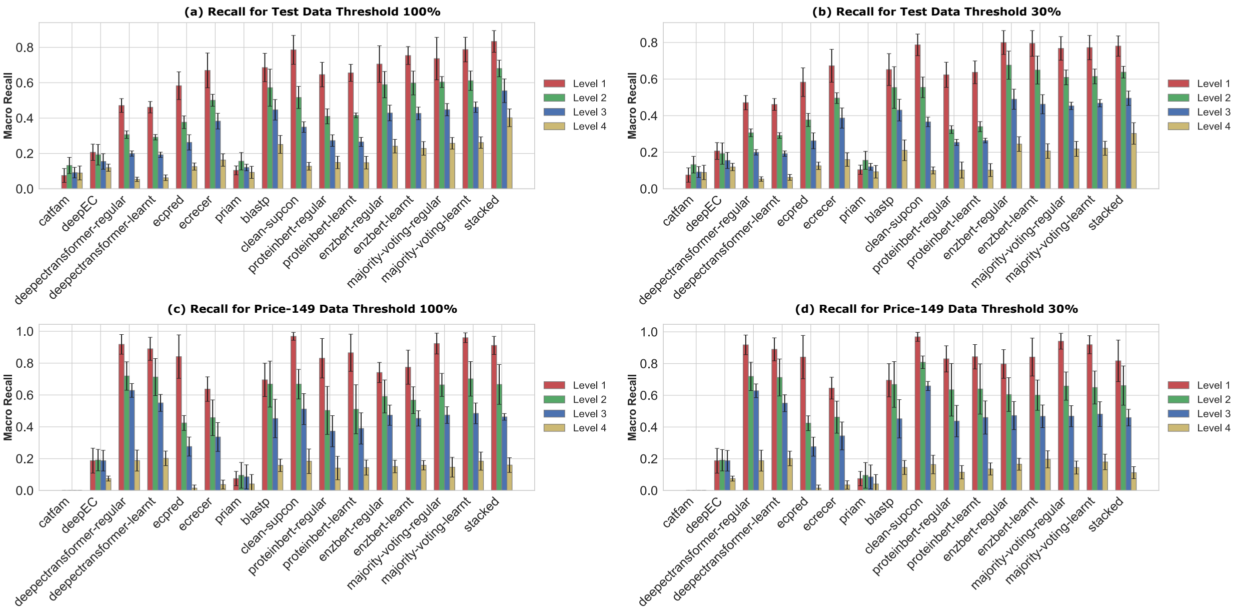

Figure S8. Macro recall for exact EC number prediction across all models and test sets.

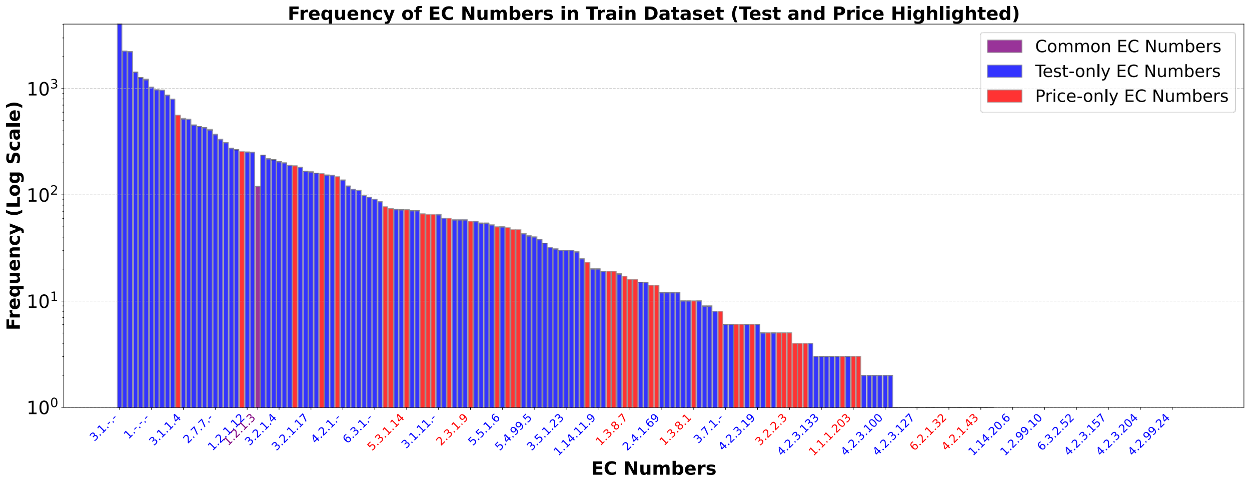

Figure S9. Frequency of EC Numbers in the Train Dataset with Test and Price-149 Datasets Highlighted: The plot displays the frequency of EC numbers from the train dataset, divided into three categories. Purple bars represent EC numbers that are common between the test and Price-149 datasets. Blue bars indicate EC numbers that are exclusive to the test dataset, while red bars represent EC numbers exclusive to the Price-149 dataset. The y-axis is displayed on a logarithmic scale to emphasize differences in frequency, and key EC numbers (common and every 5th label) are highlighted on the x-axis for clarity.

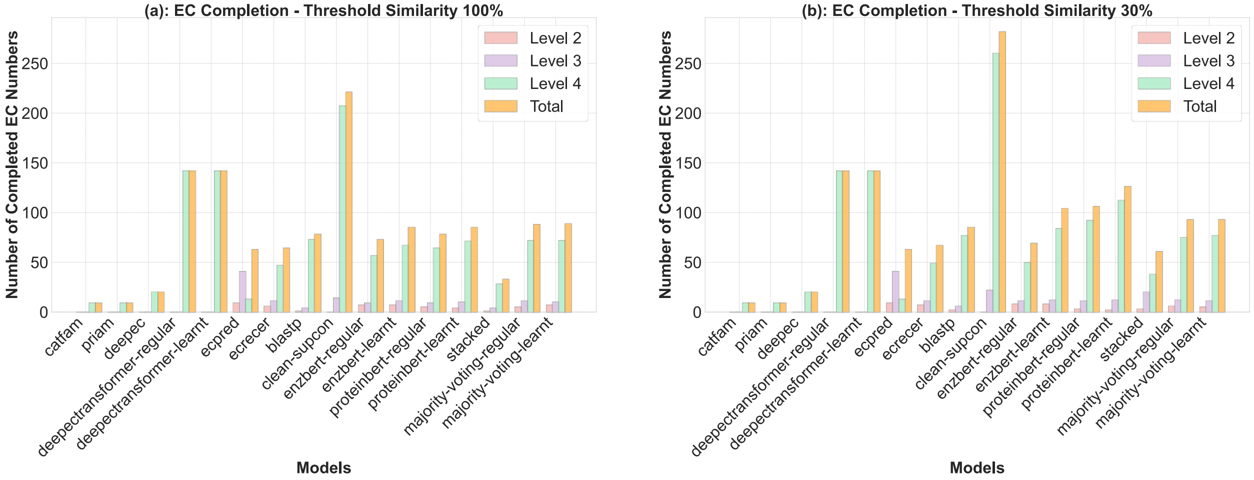

Figure S10. Comparison of models in completing EC numbers under different sequence similarity thresholds. (a) shows the number of EC numbers completed at 100% similarity, while (b) presents the results at 30% similarity. Bar heights represent completions at levels 2, 3, and 4, with an additional bar indicating the total completions per model. This visualization highlights each model's ability to provide more specific EC annotations as well as its overall performance across multiple levels of detail.

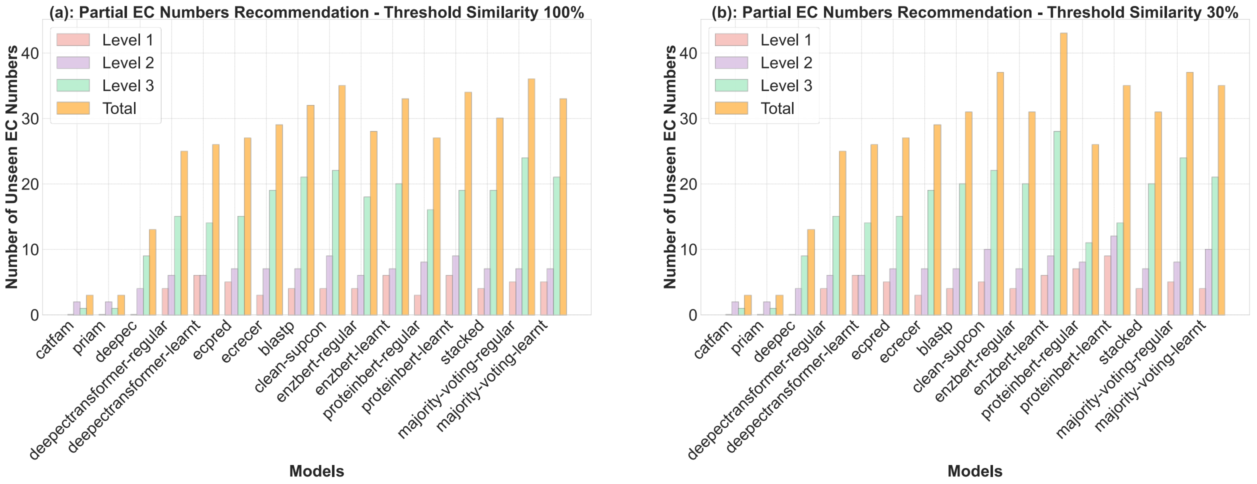

Figure S11. Partial EC number recommendation performance across models at different sequence similarity thresholds. (a) shows the number of unseen EC numbers correctly recommended by each model at levels 1, 2, and 3 for sequences with a 100% similarity threshold, while (b) presents the results for sequences with a 30% similarity threshold. Bars are grouped to represent the number of EC numbers correctly recommended at each level, with an additional bar for the total recommendations. This visualization highlights the ability of models to recommend unseen EC numbers partially.

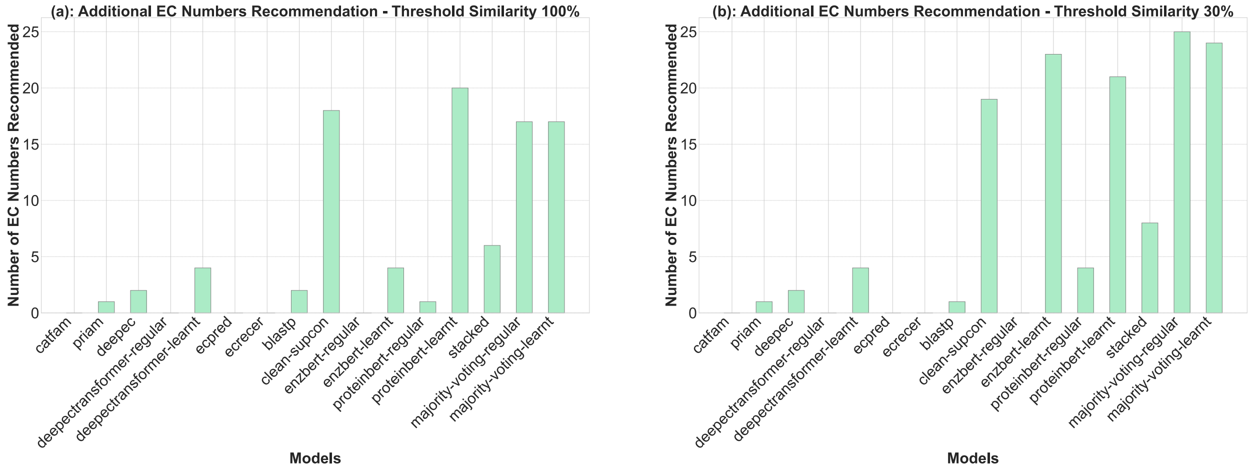

Figure S12. Comparison of "additional" EC number recommendation across models at two sequence similarity thresholds. (a) displays the number of EC numbers recommended by each model for sequences at a 100% similarity threshold, whereas (b) shows the results for sequences at a 30% similarity threshold. The analysis focuses on the models' ability to provide supplementary EC number predictions beyond the true annotations, offering insights into their capacity to identify related enzymatic functions. Each bar represents the total additional EC numbers predicted per model.

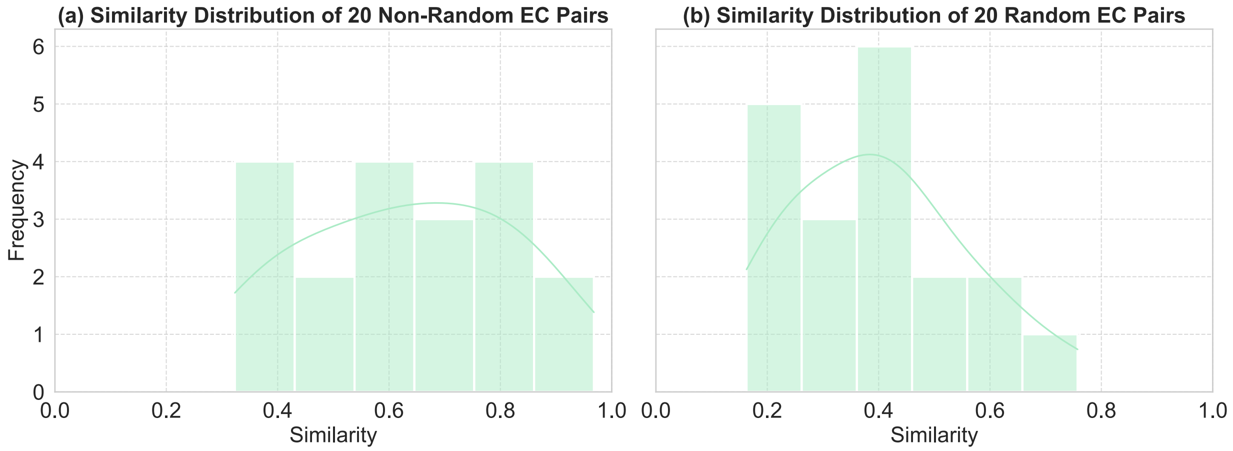

Figure S13. Distribution of pairwise reaction similarity scores for (a) 20 pairs of EC numbers from the same multi-functional enzymes, and (b) 20 pairs of randomly selected EC numbers. The results show that EC numbers from multi-functional enzymes tend to have higher similarity scores (closer to 1), while random EC pairs typically show low similarity (closer to 0).

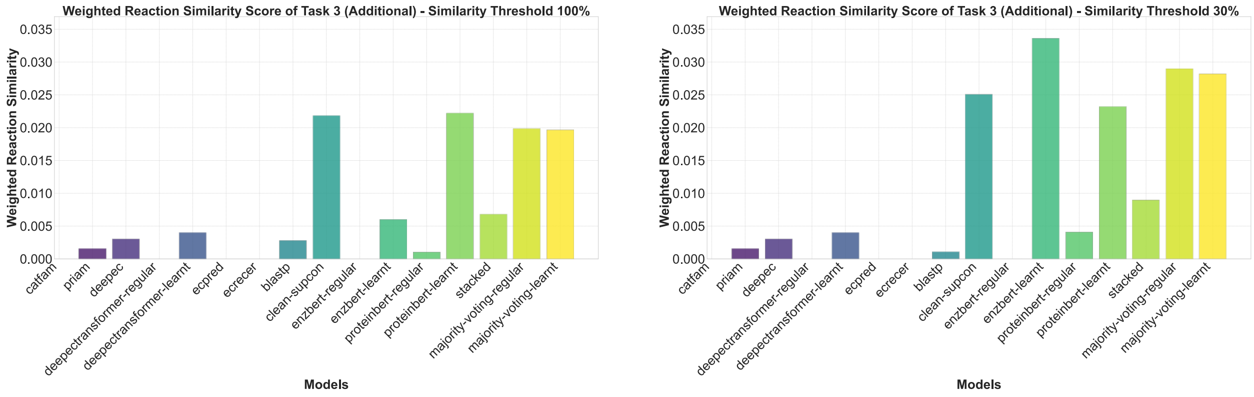

Figure S14. Weighted similarity score of models on "additional" EC number recommendation tasks at 100% and 30% sequence similarity thresholds, respectively. Each bar represents the performance of a model under the respective task and threshold.

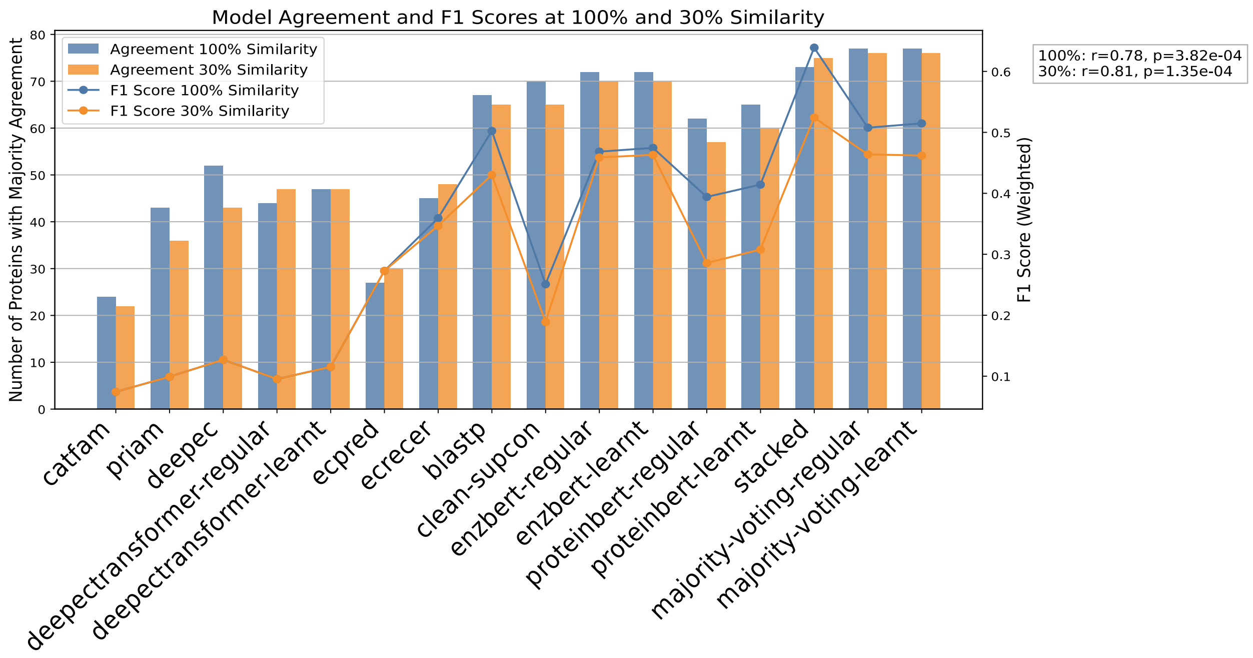

Figure S15. Comparison of model agreement on completed EC numbers and their corresponding weighted F1 scores at 100% and 30% sequence similarity. Bar plots show the number of EC numbers for which each model agrees with the majority prediction, while lines indicate the F1 score for each model. Pearson correlation coefficients (r) and p-values between agreement counts and F1 scores are shown in the top-right corner, demonstrating a strong positive relationship between model agreement and predictive performance. Pearson correlation coefficient (r) measures the linear relationship between two continuous variables. It ranges from –1 to 1, where 1 indicates a perfect positive linear correlation, –1 indicates a perfect negative linear correlation, and 0 indicates no linear correlation. The associated p-value tests the null hypothesis that there is no correlation; a small p-value (e.g., <0.05) indicates that the observed correlation is statistically significant.

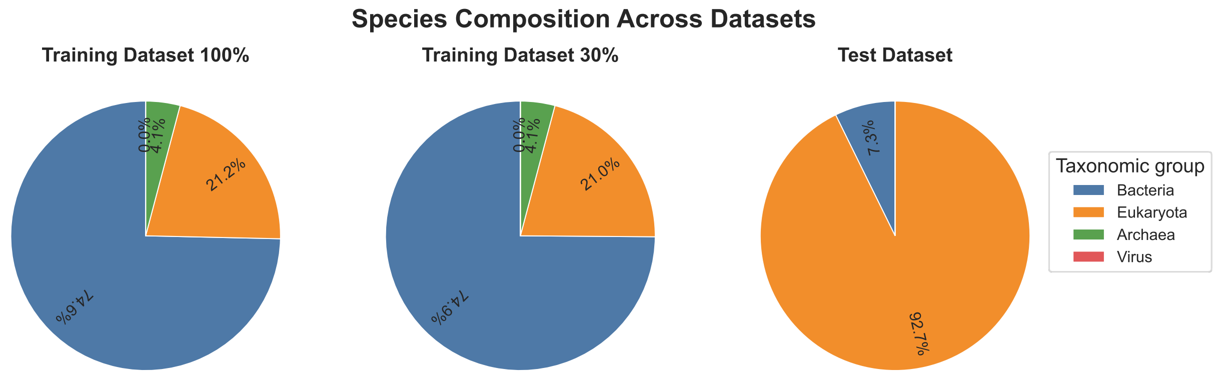

Figure S16. Circle plots showing the distribution of major taxonomic groups in the training and test datasets. The 100% training dataset contains approximately 192,872 Bacterial, 54,868 Eukaryotic (including human), 10,634 Archaeal, and one Virus (Duplodnaviria) sequence. The 30% training dataset includes 192,825 Bacterial, 54,033 Eukaryotic, 10,634 Archaeal, and one Duplodnaviria sequence. The test dataset consists of 434 Eukaryotic and 34 Bacterial sequences. Bacterial sequences dominate the training datasets, while the test set is primarily composed of Eukaryotic sequences.

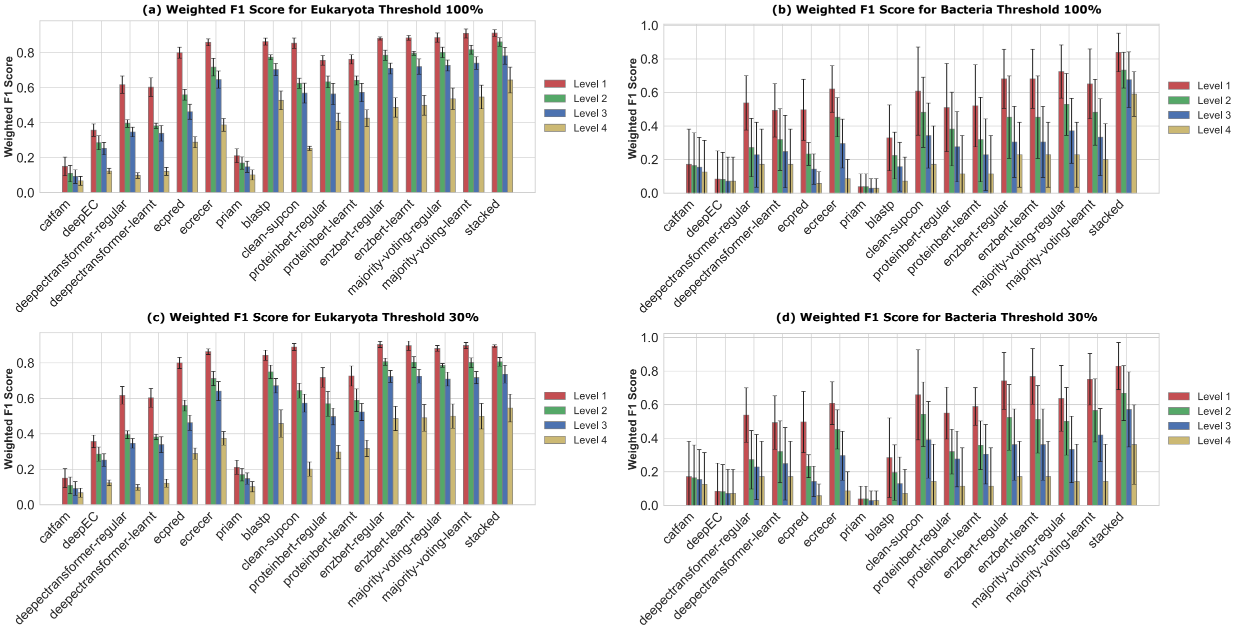

Figure S17. Weighted F1 scores with variance for enzyme commission (EC) number classification across different models and EC levels, separated by species group and similarity threshold. Panels (a) and (b) show results for Eukaryota and Bacteria at the 100% sequence similarity threshold, respectively, while panels (c) and (d) display the corresponding results at the 30% similarity threshold. Each bar represents the weighted F1 score at EC levels 1 to 4, highlighting model performance variation across taxonomic groups and classification granularity. The results demonstrate differences in model effectiveness depending on species and sequence similarity cutoff.

Supplementary Methods

Overview of EC number prediction models

In this section, we offer overviews of models used in this benchmark with publicly available software implementations. These models were selected based on their code availability, compatibility with the data, and their prominence in the literature.

1. ***PRIAM***
   In 2003, PRIAM [1] was introduced and later updated to version 2 in 2018. It functions as a profile-based model that predicts enzyme functions by employing a classification scheme found in the ENZYME database [2]. The model conducts a homology search between each protein sequence and its respective profile, resulting in a list of matches. Following this, a predefined enzyme-specific rule is applied to each ENZYME entry to determine if it fulfills the criteria, ultimately generating a list of predicted enzymes. PRIAM undergoes testing on the Swiss-Prot database [3], showcasing high precision and recall in its performance.

2. ***CatFam***
   CatFam [4], proposed in 2008, utilizes protein sequences to construct a profile database of function annotations, achieving high precision in this regard. It also excels in generating hypotheses, although with a slightly lower precision but better recall. Throughout the process of creating the database, researchers carefully monitor the adjustable false-positive rate to maintain an acceptable precision level for each profile.

3. ***BLASTp***
   BLASTp (Basic Local Alignment Search Tool for proteins) [5] is a widely used bioinformatics tool designed to compare an amino acid query sequence against a protein sequence database, identifying regions of local similarity between sequences. This tool plays a crucial role in predicting the function of unknown proteins by aligning them with known sequences, thus inferring potential functional and evolutionary relationships. BLASTp's ability to quickly search and align large databases makes it invaluable for tasks such as identifying homologous proteins, annotating genes, and understanding protein structure and function. The tool is accessible through the NCBI (National Center for Biotechnology Information) website [6], which provides both an online interface and downloadable software for more extensive local analyses.

4. ***ECPred***
   In 2018, the ECPred [7] model was proposed, offering distinct models for predicting each level of the EC number, from level 0 to level 4. This model amalgamates the outcomes of three separate predictors; each trained on the Swiss-Prot database at their respective levels. Although ECPred outperforms previous models, it is ineffective for handling multi-functional enzymes, and its performance in predicting 4-level EC numbers is suboptimal.

5. ***DeepEC***

Further progress in 2018 gave rise to the DeepEC [8], which can predict 4-level EC numbers and multi-functional enzymes. DeepEC consists of three independent but coherent Convolutional Neural Networks (CNNs): CNN-1 for enzyme and non-enzyme predictions, CNN-2 for predicting EC numbers up to level 3, and CNN-3 for predicting EC numbers up to level 4. Additionally, a homology-based predictor, BLASTp, is integrated into the model as a fallback in case CNN-2 and CNN-3 fail to predict EC numbers accurately.

6. ***ECRECer***

In 2022, a new model called ECRECer [9] has emerged in this domain. ECRECer is based on multiagent dual-core learning and boasts an exciting capability to predict the fourth level of incomplete EC numbers. ECRECer can do three tasks: 1) enzyme vs non-enzyme classification task, 2) single or multi-functional enzyme classification task, and 3) EC number prediction task. Moreover, it can provide recommendations for the top 20 EC numbers with the highest scores for an unknown enzyme.

7. ***CLEAN***

CLEAN (Contrastive Learning-Based Enzyme Annotation) [10] is an innovative model designed to enhance the accuracy of enzyme annotation by leveraging contrastive learning techniques. Unlike traditional models that rely primarily on sequence alignment or feature extraction alone, CLEAN distinguishes between similar and dissimilar protein sequences through contrastive learning. CLEAN's algorithm operates by first encoding protein sequences into high-dimensional representations using a neural network architecture. It then applies contrastive learning, a technique that involves creating pairs of similar and dissimilar sequences based on known functional annotations. The model is trained to minimize the distance between representations of sequences that share the same EC number while maximizing the distance between those that do not. This training process enables CLEAN to learn fine-grained differences between protein sequences, even when their overall sequence similarity is high.

8. ***DeepECTransformer***

DeepECTransformer [11], developed by the authors of the DeepEC model in 2023, represents a significant advancement in EC number prediction by employing the Transformer layers [12] as the neural network architecture. This model harnesses the power of self-attention mechanisms to capture complex dependencies within protein sequences, allowing it to process entire sequences holistically and identify critical features for accurate enzyme classification. By embedding protein sequences into a continuous vector space and utilizing multiple layers of self-attention and feedforward networks, DeepECTransformer focuses on the most relevant sequence regions, filtering out extraneous information. Like DeepEC, BLASTp is also integrated into the DeepECTransformer.

9. ***EnzBert***

Another Transformer-based model, introduced in 2023, is EnzBert [13]. EnzBert is based on a pre-trained language model named ProtBert-BFD [14]. ProtBert-BFD is the BERT architecture [15] pretrained on Big Fantastic Database (BFD) dataset [16] containing 2.1 billion protein sequences. EnzBert is the finetuned ProtBert-BFD on different datasets including SwissProt (release 2021_04). Model's parameters are shown in Table S2.

10. ProteinBERT

ProteinBERT [17] is a BERT-based language model pretrained on UniRef90 protein sequences and their corresponding GO (Gene Ontology) annotations [18]. UniRef90 is a clustered database of protein sequences derived from the UniProtKB (Universal Protein Knowledgebase) and selected UniParc (UniProt Archive) sequences [3]. The model architecture is composed of two interconnected components: a local part that processes protein sequences and a global part that handles the GO annotation vector associated with those sequences. These components are pretrained simultaneously, enabling the model to effectively manage sequences of varying lengths, including those of extended size. Additionally, the model's computational and memory requirements increase linearly with sequence length, making it scalable. ProteinBERT can be fine-tuned for a variety of classification tasks, ranging from protein structure prediction to the prediction of biophysical properties. Model's parameters are shown in Table S3.

Implementation

The selected models have their code available. However, DeepEC, ECPred, and DeepECTransformer do not provide their model training code, so we used their trained models instead of training them from scratch. Consequently, there is a possibility that some protein sequences from the test data were seen during the training of these models. The remaining models were trained from scratch for both the 30% and 100% similarity thresholds. The only model requiring pretraining was ProteinBERT. The pretraining and fine-tuning/training were conducted using each model's provided code. EnzBert also has a pretraining step, however, we skipped pretraining because it was expensive in terms of computation and time. ProteinBERT was pretrained and fine-tuned on a single NVIDIA Tesla A100-40GB GPU within a local cluster, named Alderaan[1], over the course of three weeks. The training and testing of other models were conducted on a single NVIDIA RTX 6000-40GB GPU. We alternated between these two clusters due to restrictions on library installations and storage limitation. Except for the homology-based models, all other models were implemented in Python. We have gathered their codes and library requirements into a unified benchmarking platform, available at https://github.com/dsaeedeh/EC-Bench.

Setting of ensemble models

We considered two ensemble models in EC-Bench: majority voting and stacking.

1. Majority voting setting

[1] https://ccm-docs.readthedocs.io/en/latest/alderaan/

For each input sequence, we collect the predictions from all models that generate at least one EC number, and the EC number with the highest frequency is selected as the final output. In cases where each predicted EC number has a frequency of 1, a random EC number is chosen as the final output. This method is particularly robust when the models tend to agree on the correct prediction, as it effectively reduces the impact of individual model errors by reflecting the consensus among the models.

2. ***Stacking setting***

In our benchmarking task, we use stacking to leverage the strengths of different models by training a meta-model on the predictions of the base models. The base models first generate their predictions independently, and these predictions are then used as input features for the meta-model, which makes the final prediction. This approach allows us to capitalize on the complementary strengths of different models, potentially enhancing the overall predictive performance. The meta-model is carefully selected and trained on a validation set to ensure that it effectively combines the outputs of the base models.

To stack models, we selected a few top-performing models to enhance the overall performance of the stacked model while also reducing training time. The selected models include DeepECTransformer, ECRECer, BLASTp, CLEAN, EnzBert, and ProteinBERT. The meta-model used for stacking is an instance of the MLPClassifier from the scikit-learn (sklearn) library [17]. The MLPClassifier, which stands for Multi-Layer Perceptron Classifier, is a type of neural network commonly employed for classification tasks. Parameters used to train the MLPClassifier is listed in Table S1.

For training the meta-model, we utilized validation data from the Swiss-Prot 2023-02 release, ensuring consistency by applying the same preprocessing steps that were used for the training data. The validation dataset consists of 19,007 sequences for the 100% similarity threshold and 18,279 sequences for the 30% similarity threshold. After evaluating the models on this validation set and train set, we merged the output from training and validation data to train the meta-model, providing it with a comprehensive and diverse dataset to learn from. This approach aimed to maximize the meta-model's ability to generalize across different EC number predictions by incorporating a wide range of sequence variations during training.

Accuracy measurement

Precision measures the proportion of correctly predicted positive labels out of all labels predicted as positive by the model. Recall, also known as sensitivity or true positive rate, measures the proportion of correctly predicted positive labels out of all actual positive labels. The weighted F1 score is a metric that combines both precision and recall into a single number, providing a balanced measure of a model's accuracy. Unlike the regular F1 score, the weighted F1 accounts for the imbalance in the dataset by considering the support (i.e., the number of true instances) of each class (i.e., distinct EC numbers). The formula for the F1 score for a single EC number class is:

$$\text{Precision} = \frac{\text{TP}}{\text{TP} + \text{FP}}$$

$$\text{Recall} = \frac{\text{TP}}{\text{TP} + \text{FN}}$$

$$\mathrm{F1} = 2 \times \frac{\mathrm{Precision} \times \mathrm{Recall}}{\mathrm{Precision} + \mathrm{Recall}}$$

Where TP is True Positives, FP is False Positives, and FN is False Negatives.
The weighted F1 score is then calculated by:

$$\mathrm{Weighted\ F1} = \frac{\sum_{i=1}^{N} (\mathrm{F1_i} \times \mathrm{support_i})}{\sum_{i=1}^{N} \mathrm{support_i}}$$

where $\mathrm{F1_i}$ is the F1 score for EC number i, $\mathrm{support_i}$ is the number of true instances for EC number i, and N is the total number of distinct EC numbers.
A high precision indicates that when the model predicts a positive EC number, it is correct most of the time, high recall means that the model predicts correct EC Numbers for most of the true positives, which is crucial in scenarios where missing a positive case is costly, and a high weighted F1 score indicates that a model performs well across both precision and recall while accounting for EC number class imbalances in the dataset.
Trade-off between precision and recall is different across different model types: while homology-based approaches offer high precision but suffer from recall loss when similarity decreases, deep learning models maintain stable recall.

Optimizing EC number prediction thresholds

The EC number prediction task is formulated as a multi-label classification problem, as a single enzyme can be associated with multiple EC numbers corresponding to different catalytic activities (i.e. multi-function). In multi-label classification tasks, deciding how to convert predicted probabilities into discrete class assignments is crucial for achieving accurate and meaningful predictions. A standard binary classification model typically applies a fixed threshold (e.g., 0.5) to determine whether a class should be assigned to a given instance. However, this approach may not be optimal for EC number prediction, where some classes may require more conservative thresholds while others benefit from more inclusive thresholds. For example, consider a neural model that uses a sigmoid activation function at the output layer and optimizes binary cross-entropy loss across all labels. The sigmoid activation function maps the output of each neuron to a probability value between 0 and 1, making it appropriate for multi-label classification tasks where each label is predicted independently. For a given input x, the sigmoid function is defined as:

$$Sigmoid(x) = \frac{1}{1 + e^{-x}}$$

This output represents the predicted probability that a specific EC number is present for the given input. Binary cross-entropy loss is commonly used in multi-label classification to evaluate the difference between the predicted probabilities and the actual binary labels (1 for presence, 0 for absence). Together, the sigmoid activation and binary cross-entropy loss allow a model to predict the presence of multiple EC numbers for a single enzyme.
However, if the model assigns probabilities of 0.45 to EC number A, 0.48 to EC number B, and 0.52 to EC number C, a uniform threshold of 0.5 would only assign EC number C, potentially

missing relevant classifications. Conversely, if a protein has multiple functional annotations with probabilities just below 0.5, a strict thresholding approach may fail to capture all relevant functions. To mitigate these challenges, we introduce a complementary strategy as a wrapper solution:

- *Learning Class-Specific Thresholds ("Learnt Models"):* Instead of applying a fixed threshold across all classes *("Regular Models"),* EC-Bench learns an optimal threshold for each EC number (class). This is done by maximizing the F1 score for each class empirically, evaluating threshold values from 0 to 1 in increments of 0.01. During prediction, these learned thresholds are used to classify an instance into a particular EC number. If none of the predicted probabilities exceed their respective learned thresholds, the model selects at least the class with the highest probability. This approach is particularly useful for rare EC numbers, where setting a higher threshold may prevent false positives, while more common EC numbers may benefit from a lower threshold to avoid missing relevant classifications.

If the highest predicted probability for a given class exceeds a high-confidence threshold (0.8), the model assigns that class.

Additional classes are considered only if their probabilities exceed a secondary threshold (0.5), allowing for multi-label classification where appropriate.

These balances ensure that the model selects confident predictions, while allowing multiple assignments when necessary.

Species-specific performance analysis

To assess possible taxonomic bias, we examined the species distribution in the training data and compared model performance between major species groups. Figure S16 shows the species composition across the training and test datasets. Bacteria dominate both training sets, while the test set contains mostly Eukaryotic sequences. To evaluate whether the imbalance affected prediction quality, we computed the weighted F1-score separately for Eukaryotic and Bacterial sequences among all models. This distribution is reflected in the weighted F1 trends for the species-specific subsets (Figure S17). The weighted F1 score trends for Eukaryotes closely follow the overall general F1 pattern observed across all species, showing consistent performance across models and EC levels. In contrast, the bacterial subset, despite having fewer test samples, exhibits a similar trend to the general F1 scores for most models. However, BLASTp performs notably worse on bacteria compared to EnzBert models, especially at finer EC levels and lower similarity thresholds.

References

[1] Claudel-Renard C, Chevalet C, Faraut T, Kahn D. Enzyme-specific profiles for genome annotation: PRIAM. Nucleic Acids Res. 2003 Nov 15;31(22):6633-9. doi: 10.1093/nar/gkg847. PMID: 14602924; PMCID: PMC275543.

[2] Bairoch A. The ENZYME database in 2000. Nucleic Acids Res. 2000 Jan 1;28(1):304-5. doi: 10.1093/nar/28.1.304. PMID: 10592255; PMCID: PMC102465.

[3] https://www.uniprot.org/uniprotkb

[4] Yu C, Zavaljevski N, Desai V, Reifman J. Genome-wide enzyme annotation with precision control: Catalytic families (catfam) databases. Proteins. 2009;74(2):449–460.

[5] Altschul, S.F., Gish, W., Miller, W., Myers, E.W. and Lipman, D.J., 1990. Basic local alignment search tool. Journal of Molecular Biology, 215(3), pp.403-410.
[6] https://www.ncbi.nlm.nih.gov/
[7] Dalkiran, A., Rifaioglu, A.S., Martin, M.J. et al. ECPred: a tool for the prediction of the enzymatic functions of protein sequences based on the EC nomenclature. BMC Bioinformatics 19, 334 (2018). https://doi.org/10.1186/s12859-018-2368-y
[8] Ryu JY, Kim HU, Lee SY. Deep learning enables high-quality and high-throughput prediction of enzyme commission numbers. Proc Natl Acad Sci U S A. 2019 Jul 9;116(28):13996-14001. doi: 10.1073/pnas.1821905116. Epub 2019 Jun 20. PMID: 31221760; PMCID: PMC6628820.
[9] Zhenkun Shi, Rui Deng, Qianqian Yuan, Zhitao Mao, Ruoyu Wang, Haoran Li, Xiaoping Liao, Hongwu Ma. Enzyme Commission Number Prediction and Benchmarking with Hierarchical Dual-core Multitask Learning Framework. Research. 2023;6: 0153.DOI:10.34133/research.0153
[10] Yu T, Cui H, Li JC, Luo Y, Jiang G, Zhao H. Enzyme function prediction using contrastive learning. Science. 2023 Mar 31;379(6639):1358-1363. doi: 10.1126/science.adf2465. Epub 2023 Mar 30. PMID: 36996195.
[11] Kim, G.B., Kim, J.Y., Lee, J.A. et al. Functional annotation of enzyme-encoding genes using deep learning with transformer layers. Nat Commun 14, 7370 (2023). https://doi.org/10.1038/s41467-023-43216-z
[12] Vaswani, A. "Attention is all you need." Advances in Neural Information Processing Systems (2017).
[13] Nicolas Buton, François Coste, Yann Le Cunff, Predicting enzymatic function of protein sequences with attention, Bioinformatics, Volume 39, Issue 10, October 2023, btad620, https://doi.org/10.1093/bioinformatics/btad620
[14] A. Elnaggar et al., "ProtTrans: Toward Understanding the Language of Life Through Self-Supervised Learning," in IEEE Transactions on Pattern Analysis and Machine Intelligence, vol. 44, no. 10, pp. 7112-7127, 1 Oct. 2022, doi: 10.1109/TPAMI.2021.3095381.
[15] Devlin, Jacob, et al. "Bert: Pre-training of deep bidirectional transformers for language understanding." Proceedings of the 2019 conference of the North American chapter of the association for computational linguistics: human language technologies, volume 1 (long and short papers). 2019.
[16] https://bfd.mmseqs.com/
[17] Nadav Brandes, Dan Ofer, Yam Peleg, Nadav Rappoport, Michal Linial, ProteinBERT: a universal deep-learning model of protein sequence and func-tion, Bioinformatics, Volume 38, Issue 8, March 2022, Pages 2102–2110, https://doi.org/10.1093/bioinformatics/btac020
[18] https://ftp.ebi.ac.uk/pub/databases/GO/goa/UNIPROT/
